# Sleeping neonates track transitional probabilities in speech but only retain the first syllable of words

**DOI:** 10.1038/s41598-022-08411-w

**Published:** 2022-03-15

**Authors:** Ana Fló, Lucas Benjamin, Marie Palu, Ghislaine Dehaene-Lambertz

**Affiliations:** Cognitive Neuroimaging Unit, CNRS ERL 9003, INSERM U992, CEA, Université Paris-Saclay, NeuroSpin Center, Gif/Yvette, France

**Keywords:** Language, Learning and memory

## Abstract

Extracting statistical regularities from the environment is a primary learning mechanism that might support language acquisition. While it has been shown that infants are sensitive to transition probabilities between syllables in speech, it is still not known what information they encode. Here we used electrophysiology to study how full-term neonates process an artificial language constructed by randomly concatenating four pseudo-words and what information they retain after a few minutes of exposure. Neural entrainment served as a marker of the regularities the brain was tracking during learning. Then in a post-learning phase, evoked-related potentials (ERP) to different triplets explored which information was retained. After two minutes of familiarization with the artificial language, neural entrainment at the word rate emerged, demonstrating rapid learning of the regularities. ERPs in the test phase significantly differed between triplets starting or not with the correct first syllables, but no difference was associated with subsequent violations in transition probabilities. Thus, our results revealed a two-step learning process: neonates segmented the stream based on its statistical regularities, but memory encoding targeted during the word recognition phase entangled the ordinal position of the syllables but was still incomplete at that age.

## Introduction

From before birth, infants demonstrate learning capacities. During the last weeks of gestation, they learn some prosodic features of their native language^1^ and their mother’s voice^2^, as the taste of the amniotic liquid^3^. A few hours after birth, they become familiar with their mother’s face^4^. Neonates also quickly adapt to repeated sensory information. For example, after a few minutes of familiarization with a word or a face, they notice when it changed^5–7^. Yung infants are also sensitive to structure founded on repetitions^8,9^ and notice second-level regularities in auditory sequences, which reveal integration capacities over periods of several tens of seconds. For instance, when presented with sequences of four repeated tones followed by a new tone, they display a mismatch response when the fifth tone is a repetition revealing that they were expecting a change ^10,11^. Despite these undeniable learning and memory capacities, very little is known about the underlying mechanisms, the information neonates are sensitive to, and the format of representation in which information is stored.

Here we focused on a primary yet indispensable fast learning mechanism: statistical learning. Statistical learning refers to the capacity to detect regularities in the input. Abundant literature^12^ shows that this mechanism is common across domains (visual, auditory)^13–17^, species (primates, rodents, dogs)^18–20^, and extends to different stimulus/scene complexity levels. Concerning language acquisition, statistical learning has been proposed as a critical mechanism to explain how infants might discover linguistic regularities. For example, it might serve to identify word candidates based on frequently co-occurring syllables^16^, to discover phonotactic and acoustic patterns^21,22^, and to detect morphological and syntactic regularities^23^.

Experimental evidence supporting the role of statistical learning in language acquisition has been mainly obtained in word segmentation tasks from an artificial speech stream in which acoustic cues have been removed. In a seminal study^16^, 8-month-old infants were first exposed to 3 min of an artificial speech (thereafter called Structured stream) constituted by four randomly concatenated tri-syllabic pseudo-words, with the drops of transition probabilities (TPs) between syllables as the only cue to word boundaries. Within a pseudo-word, the first two syllables predict the following syllable (TP equal to 1), while the last syllable could be followed by any other of the three pseudo-words (TP equal to 1/3). When test triplets were then played in isolation, infants’ looking pattern differed between the pseudo-words (i.e., Words: both TPs in the triplet equal 1) and triplets straddling a TP drop (i.e., Part-words: one TP equal 1 and the other equal 1/3). This result uncovered that infants are sensitive to the statistical relations between syllables, yet, it remains unknown what they exactly learn.

It is commonly assumed that infants segment the stream into words that are memorized and subsequently recognized when presented in isolation, assuming the extraction of word-candidates^24^. However, two other hypotheses can also explain the novelty preference for part-words. Infants may compute the transitional probabilities matrix between all syllables through synaptic plasticity and Hebbian learning^25^ without segmenting the stream^26,27^. The different association strength between syllables in Words and Part-Words could support the difference between these conditions. Alternatively, infants may segment the stream using the drop of transitional probabilities at the end of the Words but only memorize the syllable following the drop. Indeed, since this syllable is less predictable during the stream, it might induce surprise, a powerful learning factor in infants^28^. The three hypotheses are not dissociable in the existing studies since they all result in differential responses to Words and Part-words. Nevertheless, each explanation relies on different mechanisms in terms of computational complexity and neural bases.

A crucial difference between encoding the TPs matrix and segmenting the stream into Words is that memory constraints may enter into play in the latter case. When a sequence of items is memorized, each item is associated with the close items (i.e., TPs or temporal proximity) and its ordinal position within the sequence^29^. Dehaene et al.^30^ proposed a taxonomy of five levels of complexity along which a sequence can be encoded: from (1) TPs between elements, (2) chunking (grouping close elements in a unit), and (3) ordinal knowledge (the elements have an ordered position in the unit) until more abstract encoding based on (4) rules and (5) nested structures. In a very recent study in 23 adult patients with implanted electrodes who listened to an artificial structured stream containing Words (i.e., as the stream described above), the first stages of this taxonomy were explored using representational similarity analyses. The authors reported a complex picture in which different brain regions hosted different representations^31^. Some electrodes located in the superior temporal gyrus, pars opercularis, and motor cortex responded to TPs encoding. Others, located in the inferior frontal gyrus, anterior temporal lobe, and posterior superior temporal sulcus, were sensitive to ordinal position (first vs. second vs. third syllable). Finally, in the hippocampus, electrodes were sensitive to Words (chunks). This study highlights the diversity of operations and brain regions involved in processing this structured stream. Given the complex maturational calendar of the different brain structures, particularly the slow maturation of the hippocampus^32^ and frontal areas^33^, one wonders what part of these results, if not all, can be generalized to young infants. Besides, attention is notably limited at a young age, especially in neonates who sleep most of the time. Thus, we may wonder whether passive exposure might be sufficient or whether some of these computations, such as representing syllables’ ordinal position and active prediction of the next item, might not be observed during sleep. In other words, our goal was to study which levels of this taxonomy newborns possess to support language acquisition.

Previous studies have shown that neonates are at least sensitive to the first level, TPs encoding. During a long familiarization with an artificial flat stream of syllables (15 mn)^34^, tones (9 mn)^35^, and syllables with varying pitch (18 mn)^36^, a different event-related response emerged to the first syllables/tones of the Words. However, as discussed above, this result may reflect either the response to a local prediction error (i.e., TPs) or to truly individual triplets. Another study using Near-Infrared Spectroscopy (NIRS) showed a differential BOLD response to Words and Part-words following a 3.5 min familiarization with a structured stream^37^. While adding that neonates can remember the extracted information for a few minutes, it leaves pending the information they retained that triggered the differential response.

**Figure 1:**
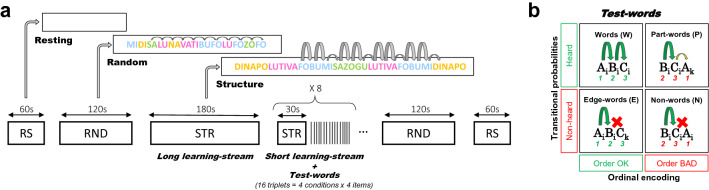
Experimental protocol. (**a**), The experiment started with a resting state period (1 mn), followed by a random stream (2 mn) then the learning stream was presented (3 mn). This long Structured stream was followed by a test phase including 8 blocks of 16 triplets presented in isolation with a jittered ISI (2–2.5 s). Each block was separated from the next by short Structured streams (30 s) to maintain learning. A Random stream and a Resting state period were again recorded at the end of the experiment to control for the effect of time, and notably infant vigilance, on EEG recordings. (**b**), Possible types of test words. Test words could have violations in the TPs between the second and third syllables, in the ordinal position of the syllables (1 2 3 vs. 2 3 1), or both.

We, therefore, proposed to investigate statistical learning in neonates further using high-density electroencephalography (EEG) (128 electrodes) in a paradigm, close to the seminal Saffran et al.’s study, i.e., based on three minutes of exposure to a Structured stream (Long learning stream), followed by the presentation of isolated triplets. Because to obtain ERP, we need many more trials than in behavioral studies, we interspersed short structured streams (30 s) between blocks of 16 isolated words to maintain learning (Fig. 1). Our goal was double, first, to describe the learning curve during the stream exposure thanks to neural entrainment, and second, to characterize the format of the learned representation by presenting four different types of triplets.

Thanks to its temporal sensitivity, EEG allows monitoring learning, even in non-participating subjects, such as sleeping neonates. In particular, in this paradigm, where syllables have a fixed duration, the auditory response induced by the regular presentation can be captured as entrainment at the stimulation frequency (f = 1/syllable duration). Crucially, this steady-state response is not limited to low-level features like syllable onset but can reflect any regular pattern the brain is tracking^38–42^. Thus, if the listener detects the 3-syllabic pattern embedded in the stream, entrainment should also be observed at the triplet frequency (1/3 of the syllabic rate). Performing an analysis in the frequency domain has many advantages relative to ERP. The steady-state nature of the neural response makes the entrained frequencies predictable (here 1/syllable duration and 1/word duration limiting the statistical analyses to these two frequencies), while the timing of the ERP is usually unknown. Moreover, by using neural entrainment, the streams can be continuous (without pauses between syllables), syllables can have a duration more compatible with natural language, and baseline issues for the computation of the ERPs during the streams are avoided^34–36^. In this regard, interpreting ERP of a continuous speech is challenging because the voltage is lower with a fuzzier onset for each syllable compared to syllables preceded by even a brief silence, and because the rapid succession of the syllables prevents a proper analysis of the responses to each syllable as late responses to one syllable and early responses of the next overlap. Therefore, we quantified the entrained neural responses at the syllabic and word rates measuring an enhanced Power and Inter Trial Coherence (ITC) during the presentation of the Structured stream and compared their values to the same variables obtained in a Random stream (random concatenation of the syllables), and Resting-state periods (i.e., without stimulation). We expected similar entrainment at the syllabic rate for the Structured and Random streams relative to resting-state, but an increased activity at the word rate during the Structured streams. The Resting-state periods and Random streams sandwiched the learning stream and test phases to control for changes in infants’ vigilance state during the recording session (Fig. 1a).

While neural entrainment at the word frequency reflects that the neonates extract the regularities in the stream, it can result from two different processes, in the same way as for the ERP differences reported in the studies discussed above^34,35^: either the neonates react to a local drop in TPs, or they recognize the re-occurrence of each triplet. To test what they learn and memorize, we compared the ERPs to isolated triplets in a post-learning phase. During this phase, 128 triplets (Test words) were presented in 8 blocks (16 triplets per block) separated by silences (2 to 2.5 s). Each block was preceded by a short learning stream (30 s) that served as re-familiarization to prevent progressive forgetting of the initial transitions probabilities between syllables caused by the presentation of Test words, half of which were inconsistent with the initial learning (Fig. 1a).

We build four types of triplets to disentangle different hypotheses on the encoding format of the retained pattern (Fig. 1b, and Table 1). We contrasted: (1) triplets respecting, or not, TPs between syllables, and (2) triplets violating, or not, the ordinal position of the syllables. Therefore, we presented the classical conditions: Words (*A*_*i*_*B*_*i*_*C*_*i*_) corresponding to the pseudo-words present in the stream, and Part-words (*B*_*i*_*C*_*i*_*A*_*k*_) corresponding to triplets straddling a TP drop. Note that in Part-words, syllables, notably the first, are not at the correct position but the initial TP is correct (TP = 1 for AB and BC). To these common conditions, we added two other conditions: Edge-words and Non-words. Edge-words (*A*_*i*_*B*_*i*_*C*_*k*_) were triplets in which the last syllable between two Words was exchanged; thus, they retained the ordinal position of the syllables, but they were never presented in the stream (last TP equaled zero). Non-words (*B*_*i*_*C*_*i*_*A*_*i*_) were triplets in which the first syllable appeared in the last position; thus, all syllables belonged to the same Word, but the ordinal position was incorrect, and the triplet was never heard (last TP equaled zero).

**Table 1:** Stimuli.

List	A_i_B_i_C_i_	A_i_B_i_C_k_	B_i_C_i_A_k_	B_i_C_i_A_i_
A	Dinapo	Dinava	napolu	napodi
	Lutiva	Lutimi	Tivafo	Tivalu
	Fobumi	Fobugu	Bumisa	Bumifo
	Sazogu	Sazopo	Zogudi	Zogusa
B	Napolu	Napofo	Poluti	Poluna
	Tivafo	Tivasa	Vafobu	Vafoti
	Bumisa	Bumidi	Misazo	Misabu
	Zogudi	Zogulu	Gudina	Gudizo
C	Poluti	Polubu	Lutiva	Lutipo
	Vafobu	Vafozo	Fobumi	Fobuma
	Misazo	Misana	Sazogu	Sazomi
	Gudina	Guditi	Dinapo	Dinagu

Triplets for each condition (Words, Edge-words, Part-words, and Non-words) used for each of the three lists. Note that Words, Part-Words, and Edge-words are swapped between lists (Non-words and Words share the same syllables) to control for any acoustic differences between conditions. One list was randomly selected for each participant.

If neonates segment the stream and encode ordinal information or at least the first syllable of a word, we expected an early differential response between *ABx* (Words and Edge-Words) and *BCx* triplets (Part-Word and Non-Words). Note that any difference before the third syllable can only be due to the encoding of the first syllables or to the first expected transition AB—*A*_*i*_*B*_*i*_ and *B*_*i*_*C*_*i*_ had both TPs equal to one. By contrast, if the response to the isolated triplets only depends on the adherence to the statistical structure of the Structured stream, the ERPs between never heard triplets (Edge-words and Non-words) and those present in the stream (Words and Part-words) should differ from the third syllable. For the sake of completeness, we also considered that memory encoding following segmentation might be sensitive to the temporal proximity of the elements belonging to the same chunk as a community structure, predicting that Non-Words (*B*_*i*_*C*_*i*_*A*_*i*_) are closer to Words (*A*_*i*_*B*_*i*_*C*_*i*_) than Part-Words (*B*_*i*_*C*_*i*_*A*_*k*_).

To summarize, stream segmentation should be revealed by neural entrainment at the word rate. Note that TP learning can be observed without stream segmentation^26^. Simple TP learning should result in a difference between triplets present or not in the stream (Words + Part-words vs. Edge-words + Non-words) and Word-recognition in a difference between *ABx* and *BCx* sequences in the subsequent test phase. The granularity of the memory encoding can be further investigated by comparing Words vs. Edge-Words and Non-words vs. Part-Words.

Additionally, we tested 32 adult participants in a behavioral online experiment analog to the infant task. After familiarization with the structured stream, participants had to rate their familiarity with the Test words. Because the stimuli (duration of the Structured streams and number of tests words) were the same as in the neonates’ study, this experiment provides a reference point of what mature and expert participants encode and memorize.

## Results

### Neural markers of learning in neonates: familiarization phase

**Figure 2:**
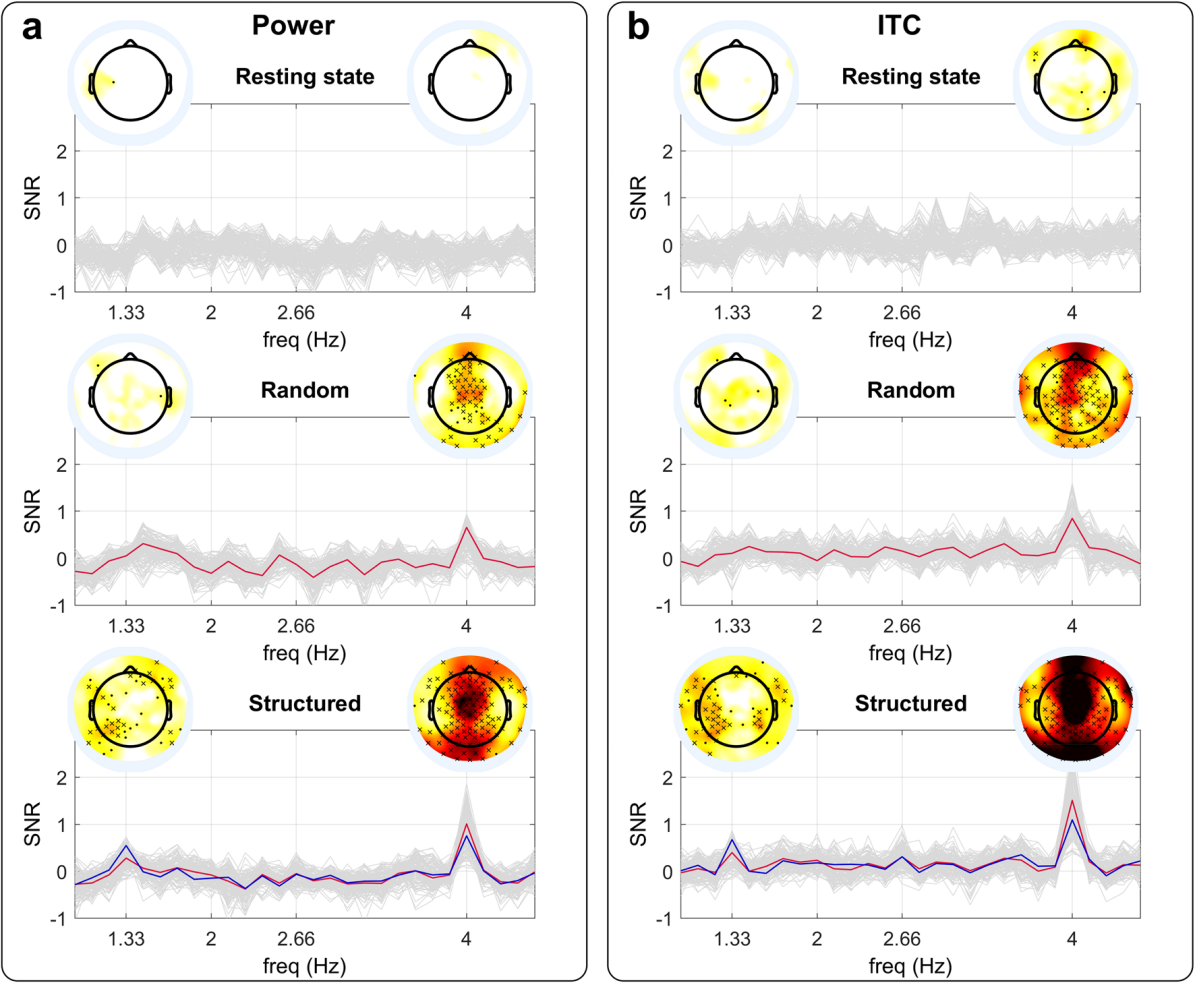
Neural entrainment to the syllabic rate (4 Hz) and the word rate (1.33 Hz) during the three periods (Resting state, Random stream, and Structured stream). (**a**), SNR for the power. In light gray, the entrainment for all electrodes. In red, the mean over the electrodes showing significant entrainment (p < 0.05, one-sided t-test, FDR corrected) at the syllabic rate. In blue, the mean over the electrodes showing significant entrainment (p < 0.05, one-sided t-test, FDR corrected) at the word rate. The topographies represent the entrainment in the electrodes space at the word rate and at the syllabic rate. Asterisks indicate the electrodes showing enhanced neural activity (cross: p < 0.05, one-sided t-test, FDR corrected; dot: p < 0.05, one-sided t-test, without FDR correction). (**b**), Same as (**a**) for ITC.

During Resting-state, as expected, no entertainment was seen either at the syllabic (4 Hz) or word (1.33 Hz) rates. As expected, for Random streams, we observed enhanced activity at the syllabic rate for many central-frontal and posterior electrodes (*p* < *0.05*, FDR corrected) and no enhanced activity at the word rate. During the Structured streams, we observed a similar enhanced oscillatory activity at the syllabic rate but also significant neural entrainment at the word rate mainly over left temporal electrodes (*p* < *0.05*, FDR corrected) (Fig. 2).

**Figure 3:**
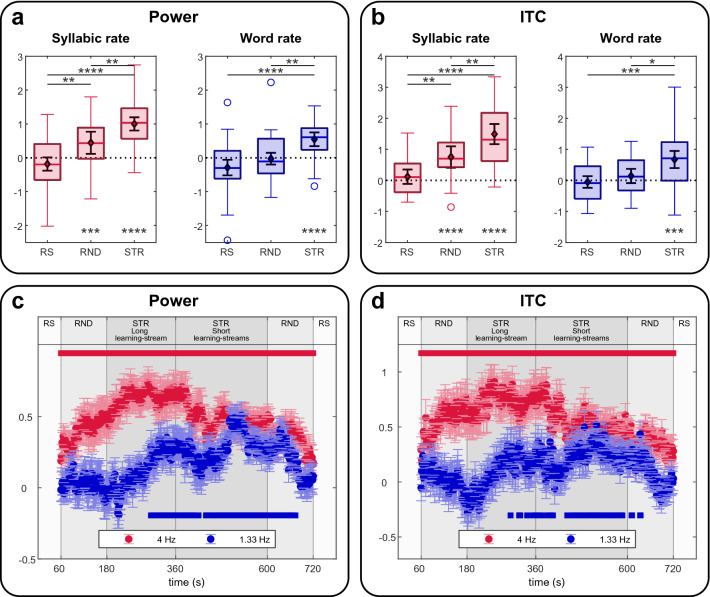
Neural entrainment. (**a**), SNR for the power at the Syllable and Word rate during the three conditions (RS = resting state, RND = random stream, STR = structured stream). Asterisks represent Bonferroni corrected p-values *** < 0.001, ** < 0.01 * < 0.05. (**b**), Same as (**a**) for ITC. (**c**) Time course of entertainment based on power computed on 120 s time windows. The times on the x-axis correspond to the center of the time windows. Error bars represent standard errors. The red line on the top indicates when the power at the Syllabic rate (4 Hz) was larger than the null hypothesis 0 (p < 0.05, one-sided t-test, corrected by FDR). The blue line on the bottom indicates when the power at the Word rate (1.33 Hz) was larger than the null hypothesis 0 (p < 0.05, one-sided t-test, corrected by FDR). (**d**), Same as (**c**) for ITC.

As a supplementary analysis, we compared the entrainment at each target frequency over the electrodes showing an enhanced response on any of the conditions, using a 1-way-ANOVA with condition (Resting state vs Random stream vs Structured Stream) as a within-subject factor (Fig. 3a,b). Similar results were obtained for power and ITC. A main effect of condition was observed at the syllabic rate (power: *F*(2,58) = 21.8,* p* = 8.6 × 10^–08^, ITC: *F*(2,58) = 21.8,* p* = 8.7 × 10^–8^, driven by a lower power/ITC during Resting than Random (power: *p* = 0.0021, ITC: *p* = 0.0085) and Structured (*p* = 8.4 × 10^–9^, ITC: p = 7.5 × 10^–9^), and lower power/ITC during Random than Structured (power: *p* = 0.0075, ITC: *p* = 0.0017). At word rate there was a main effect of condition (power: *F*(2,58) = 10.7,* p* = 0.00018, ITC: *F*(2,58) = 8.2,* p* = 0.000706), due to a higher power/ITC during Structured than Resting (power: *p* = 2.9 × 10^–5^, ITC: *p* = 0.00038) and Random (power: *p* = 0.0052, ITC: *p* = 0.013). For the post-hoc tests, all p-values were Bonferroni corrected for multiple comparisons.

To quantify learning through the experiment, we measured entrainment at the syllabic and word rate in sliding time windows of 2 min with a 1.5 s step by concatenating the data from all conditions. For visualization of the time course of the effect, we assigned to each time window the time corresponding to its central time (e.g., time 60 s corresponds to the first time window, 61.5 to the second). Notice that because the integration window is two minutes long, the entrainment during the first minute of random, for example, includes data from the structured stream. We used a two-minute time window because while a shorter time window would provide better resolution, it would not ensure enough frequency resolution and signal-to-noise ratio^40^. Results show an increase in Power and ITC at the word rate at around 2 min from the beginning of the structured stream (Fig. 3c,d).

### Word recognition in neonates: post-learning phase

**Figure 4:**
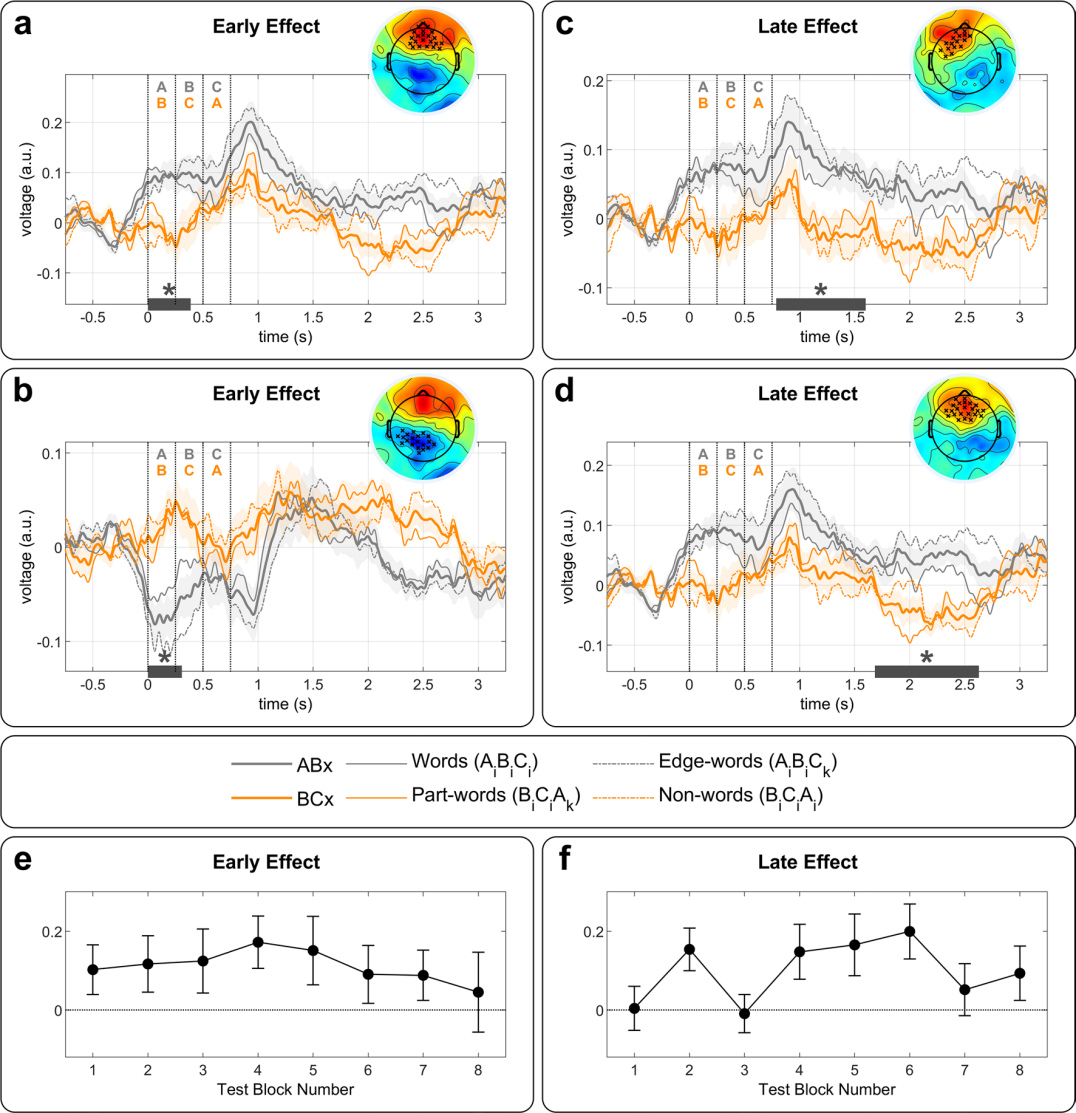
Responses to triplets in isolation. (**a**) Grand-average response to ABx and BCx triplets over the early frontal positive cluster (*p* = 0.0152) obtained from the cluster-based permutation analysis. The thick lines correspond to *ABx* (gray line) and *BCx* (orange line) conditions and the thin lines to the sub-conditions (Words and Edge-Words vs. Part-words and Non-words). Shaded areas correspond to the standard error across neonates. The time zero corresponds to the onset of the test word. Vertical lines signal the onset of each syllable and the end of the word. The topography shows the difference *ABx*-*BCx* during the time window where significant differences were observed (gray line under the plot). (**b**) Same as (**a**), but for the early negative cluster over left-temporal posterior electrodes (*p* = 0.0324). (**c**) Same as (**a**), but for a late positive cluster over frontal-left electrodes (*p* = 0.0142). (**d**) Same as (**a**), but for a later positive cluster over frontal electrodes (*p* = 0.0020). (**e**) Time progression of the ERP early effects (**a,** and **b**) over the 8 test blocks. (**f**) Time progression of the average of the two late ERP effects (**c**, and **d**) over the 8 test blocks.

We first looked for ERPs components related to ordinal position violations by comparing *ABx* (Words and Edge-words) vs. *BCx* triplets (Part-words and Non-words). A non-parametric cluster-based permutation analysis^43^ revealed a significant early difference before 500 ms in a positive frontal cluster (*p* = 0.0152, time window [0, 388] ms) and in a left-posterior negative cluster (*p* = 0.0324, time window [0, 308] ms) corresponding to the positive and negative pole of the same dipole response (Fig. 4a,b). Each syllable was 250 ms long. Thus, given the time window, this effect can only be related to recognizing the first syllable (i.e., ordinal encoding). A second difference was also observed after the offset of the triplet, in a frontal-left positive cluster (*p* = 0.0142, time window [788, 1600] ms), and even a third one later in a frontal cluster (*p* = 0.002, time window [1684, 2628] ms) (Fig. 4c,d).

We then looked for ERPs components related to TPs violations by comparing heard triplets (Words *A*_*i*_*B*_*i*_*C*_*i*_ and Part-words *B*_*i*_*C*_*i*_*A*_*k*_) vs. non-heard triplets (Edge-words *A*_*i*_*B*_*i*_*C*_*k*_ and Non-words *B*_*i*_*C*_*i*_*A*_*i*_), but we found no significant difference (*p* > 0.1). In addition, no significant differences were detected in the comparisons Words vs. Edge-words, and Part-words vs. Non-words (*p* > 0.1).

To ensure that the differential response was present from the beginning of the test phase and was not triggered by hearing isolated triplets (i.e., from the first Test-block infants might infer that three-syllable pseudo-words constituted the stream), we computed the effect throughout the eight test blocks. Specifically, we computed the differential response between ABC and BCA triplets over the electrodes and time windows where the cluster-based permutation analysis showed significant differences. Despite fluctuations likely due to the small number of trials, the effect was present from the earliest test blocks (Fig. 4e,f), suggesting that the encoding of the first syllable in Words had emerged during the long Learning stream.

### Word recognition in adults

**Figure 5:**
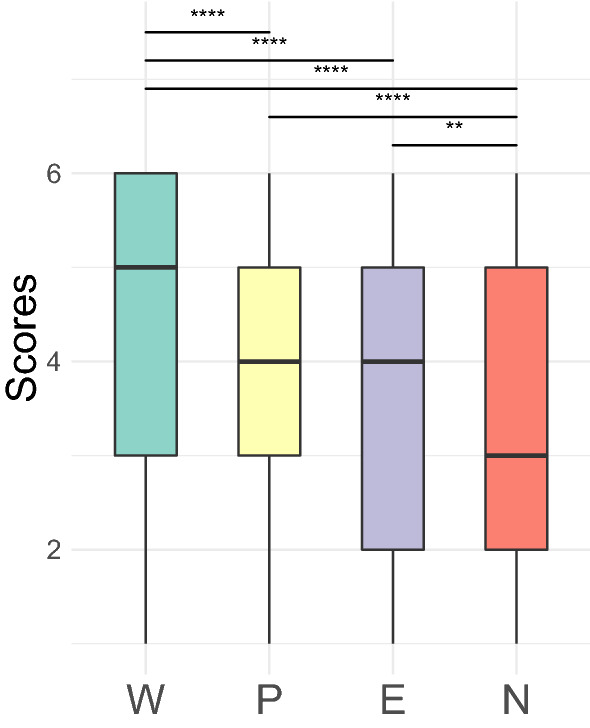
Adult behavioral experiment. The distribution of the scores for all trials and participants are represented per condition. Asterisks represent p-values of the comparisons done two by two (Tuckey tests) *** < 0.001, ** < 0.01. W = Words (*A*_*i*_*B*_*i*_*C*_*i*_), P = Part-Words (*B*_*i*_*C*_*i*_*A*_*k*_), E = Edge-Words (*A*_*i*_*B*_*i*_*C*_*k*_), N = Non-words (*B*_*i*_*C*_*i*_*A*_*i*_).

Adults rated their familiarity with the triplets on a scale after familiarization with identical streams as neonates (Fig. 5). Results from a linear mixed model using the scoring as the dependent variable, the triplet condition as a predictor, and subjects as a random factor (*Scoring* ~ *Cnd* + *1|Sbj*) showed a main effect of condition (*F*(3,3721) = 79.72,* p* < 2.2 × 10^–16^). A posthoc Tukey test revealed that the Words score was higher than each of the other conditions (*ps* < 0.0001), whereas the Non-words was the lowest, significantly inferior to Part-words (*p* < 0.0001), and to Edge-words (*p* = 0.0045). Thus adults remembered the whole words and were somewhat sensitive to ordinal position as reported by previous work^31,44^. Indeed, Edge-words, which have all syllables at the correct ordinal position but TP equals 0 for the transition between the second and third syllables, were judged as familiar as Part-words (TP are 1 and 0.33 for Part-words, and 1 and 0 in Edge-Words). Edge-words were also found more familiar than Non-words, triplets in which all ordinal positions are violated but membership to the same chunk retained.

## Discussion

Here, we used a classical speech segmentation task^16^ to investigate statistical learning in neonates. While previous studies have shown that infants are sensitive to statistical regularities in speech since birth^34,35,37^, it was still unknown what information they tracked and retained. First, our study revealed that sleeping neonates responded rapidly (within 2 min) to the tri-syllabic pattern. Second, when isolated triplets were presented, a differential response was observed from the first syllable, revealing that they expected triplets to start with a specific set of syllables. Third, TP violation did not modulate ERP to triplets. This result indicates a memory representation that no longer depended on TPs, despite TP being used to segment the stream, suggesting a switch to a different representation format.

### Learning based on TPs

The significant increase in power and ITC to word rate in the Structured stream demonstrated that TP computations lead to stream structuring. Learning occurred within 2 min of familiarization. This rapid learning is consistent with the length of the stream previously used in behavioral experiments in 8-month-old infants^16^ and EEG experiments in adults and 6-month-old infants^40,41^. Bosseler et al., reported a change in ERP depending on the syllable position from the forth minute on in neonates^36^. The concordance of learning rate across ages indicates that statistical learning abilities do not improve markedly with age, a remarkable observation given the significant maturational changes in auditory/linguistic regions and hippocampus during the first years of life^45,46^.

We did not characterize the neonates’ sleep stages. However, their general behavior during the recording session (eyes closed, hypotonia), the duration of the experiment, and the lack of task and reward, combined with the short awake periods outside of feeding in the days after birth, certainly did not favor an attentive and focused listening of the auditory input. Neonates’ success in extracting the regularities is congruent with adult studies showing neural entrainment at the word rate even when participants are distracted by a primary task^40,41^, revealing the automaticity of TP calculations.

In adult experiments, the word rate entrainment is accompanied by decreased syllabic rate entrainment^41^. Our results revealed a more complex pattern. The syllabic rate entrainment increased at the beginning of the Structured stream and decreased when word rate entrainment became significant. The initial increase entrainment at the syllabic rate might reflect stronger activation of the language network during the uncovering of the structure compared to random syllable presentation. This hypothesis would be consistent with an adult functional magnetic resonance imaging (fMRI) experiment showing that activity in the left-temporal cortex is modulated by the level of complexity of speech sequences^47^. The subsequent decrease might result from top-down inhibition of the syllabic response once the stream has been segmented.

While neural entrainment demonstrated that infants were sensitive to the rhythmic structure of the stream, this might result from an automatic error response elicited by the unpredictability of the first syllable (TPs) or by a neural response to tri-syllabic chunks (segmentation).

### Memory representation of the segmented words

ERPs to the isolated triplets revealed the format of the retained information. ERPs differed from the first syllable between *ABx* triplets (Words and Edge-Words) and *BCx* triplets (Parts-Words and Non-Words); thus, before any TP violation (*AB* and *BC* transitions were both equal to 1). Additionally, we observed no specific ERP component after a TPs violation, that is to say, between Words and Edge-Words on one side and Part-Words and Non-Words on the other side. It is important to note that in Non-words, the first syllable was presented at the last position without evoking a particular response (i.e., a difference with Part-Words). The absence of a distinctive response to the first syllable at the wrong position favors the hypothesis that it is not a particular familiarity with this syllable due, for instance, to its unpredictability during the stream, which caused the difference between *ABx* and *BCx* triplets but the ordinal position of the first syllables.

Two approaches have been proposed for flat continuous speech segmentation. From one perspective, the TPs are computed, and the drops in TPs serve as cues to word boundaries^16^. From another perspective, recurrent chunks of co-occurring syllables are identified and stored in memory^48^. Our experiment did not attempt to disentangle these two mechanisms. However, the lack of difference between heard and un-heard triplets revealed that neonates retained neither the full TP matrix nor the entire Words. Instead, they remained limited to some expectations concerning the beginning of the words. Rigorously, three options could explain a difference between *ABx* and *BCx* triplets: neonates recognize (1) that words start by one of the four *A* syllables (i.e., *Axx*), (2) the *AB* transitions, or (3) that words have a *B* in the middle position (i.e., *xBx*). Hypotheses 2 and 3 derive from considering that *B* acquires a “special status” by functioning as anchor syllables during TPs computations because they are flanked by TP = 1, meaning they establish the link between *As* and *Cs* (*A* is linked to *B* and *B* to *C*). Hypothesis 2 implies an asymmetric TP learning of the TPs flanking *Bs* (i.e., better learning of the forward TP *P(B|A)* than the backward TP *P(C|B)*). Hypotheses 1 and 3 imply segmenting the stream and relying on syllable order (i.e., what is first or second). Since the early effect we observed appears during the first syllable, it suggests that the effect concerns the first element (hypothesis 1), not the transition (hypothesis 2) or the second syllable (hypothesis 3), which should have delayed the difference until some part of the second syllable was perceived (i.e., after 250 ms). Even if coarticulation might have blurred the exact onset of the second syllable, and high-pass filtering issues might have slightly spread the effect, the difference was unequivocally present during the first syllable (Fig. 4). Moreover, there is no reason to learn better a backward transition *AB* than a forward *BC* transition unless infants are segmenting the stream, and thus, learning that words start by AB and not only the recognizing the transition. Additionally, remembering that *Bs* are the central element of the Words is not consonant with previous studies showing better encode of elements at the edges of a sequence^49^. It could be argued that infants encode that words should not start by *Bs* (i.e., ~ *Bxx*), but the complexity of this encoding makes it unlikely. Based on these considerations, we favor hypothesis 1, i.e., neonates expected the first syllable to belong to a specific set of 4 syllables.

Meanwhile, adults scored Words as highly familiar, Edge-words as more familiar than Non-words, and finally Edge-Words and Part-words as equally familiar (although Edge-words never appeared in the stream, the ordinal position of the syllables was correct). These results suggest that adults memorized the complete Words, and that they represent both TPs and ordinal position, in agreement with other recent studies^31,44^.

Altogether, our results suggest a multistep process. First, segmentation occurred either because the drop in TP produced a prediction error that singularized the non-predicted syllable (i.e., the *A* syllables) or because syllables within words become increasingly associated (around *B* syllables), leading to boundaries at the lower points of this associative landscape. In a second step, the segmented triplets are stored in memory. The memory system is probably less bounded to TPs and also relies on positional coding; however, word recognition is incomplete due to memory limitations at birth at the encoding or retrieval stage.

### Word memorization is incomplete in neonates

Neonates are thus memorizing the first syllable of the chunk (A) or eventually also the first transition (AB), pointing to an ordinal encoding, the third level of complexity in Dehaene et al. taxonomy^30^. However, they did not distinguish Words (*A*_*i*_*B*_*i*_*C*_*i*_) and Edge-words (*A*_*i*_*B*_*i*_*C*_*k*_), suggesting that neonates’ words memory was not complete. A limited memory capacity in neonates for middle positions has already been described. A NIRS study in neonates showed a better encoding of the syllables at the edges of a six-syllable pseudo word than in intermediate positions^49^. Unfortunately, the conditions in that study do not allow disentangling if the effect was due to better encoding of the first, the last, or both syllables. The recognition of bi-syllabic pseudo-words from a new pseudo-word presented two minutes later^5,6^ and of words conforming a structured stream^37^ in previous studies might have also relied on incomplete memory of the words. Even if memory is limited due to age or sleep, these results reveal that neonates store word-forms in a longer memory than an echoic buffer.

Our results demonstrate that sleep does not inhibit neonates from learning the stream regularities as it does seem to inhibit rule learning in some circunstances^11^. However, our results leave open the origin of the memory limitation we observed here, which might be due either to immaturity or to sleep. Sleep is primarily considered as consolidating memories, and while learning is suppressed during deep non-REM stage in adults, implicit learning is present during REM sleep^50^. Furthermore, infants have a very different sleep organization. Cycles are shorter with only two clear states, quiet (~ 40% of the cycle at birth) and active sleep (50–60% of the cycle at birth, equivalent of REM sleep at later age) and some intermediate state. Furthermore, micro-arousal periods occur within and between sleep states^51^. As tasks started during wake can continue during REM sleep in adults^50^, the neonatal organization of sleep may not be a limiting factor here, but this question should be further explored.

### Putative underlying neural networks

While EEG has an excellent temporal resolution, it does not provide accurate spatial resolution and information regarding the activity of brain structures. However, we may speculate from the adults’ results and the few brain imaging studies in infants investigating the maturation of the pertinent brain regions. Henin et al.^31^ isolated three main networks in a similar task in epileptic patients that might already be at work in neonates. The superior temporal region, which might be related to local processes involved in TP computations, and two memory structures: the dorsal linguistic pathway supporting verbal working memory, and the hippocampus, recently reported as engaged in sequence learning^52,53^. Although these two structures have been considered immature in infants, fMRI has revealed that they support cognitive functions in the first trimester. Notably, whereas the superior temporal regions are affected by the immediate repetition of a sentence^54^, repetition at a longer time-scale of 14 s produces activation in the inferior frontal gyrus in three-month-old infants^55^. Moreover, a NIRS study in sleeping neonates revealed that a correlated activity between left-temporal and left-frontal regions, compatible with activation in the dorsal linguistic pathway, is crucial for word learning^56^. As for the hippocampus, activity has been reported in infants as young as 3-months when performing a visual sequence learning task, with no modulation by infant’s age^57^. Thus, future work should investigate whether hippocampal circuits considered fundamental to SL, such as the monosynaptic pathway, are involved in such a word-learning task since birth. fMRI in infants might help determine how the network highlighted in adults^31^ is similarly involved in infants to support the two stages we have isolated, the relative role of the hippocampus and the linguistic network.

Before concluding, we would like to point to the accuracy of consonant encoding in newborns, which allows them to keep the relationship between 12 syllables and memorize a set of 4 first syllables despite common vowels at different ordinal word positions. This observation is not trivial given the common assumption that infants are initially limited to the most stable units, such as vowels. For example, Benavides et al.^5^ reported a larger novelty response when changing the vowels of a bi-syllabic word (e.g., *lili* to *lala*) compared to a change of consonants (e.g., *lili* to *titi*). However, a recent EEG study showed that phonetic features were at the basis of speech perception in 3-month-old pre-babbling infants, offering the possibility of a structured combinatorial code for speech analysis not limited to vowels^58^.

To conclude, despite their unquestionable immaturity, neonates reveal sophisticated learning abilities. From drops in TPs, they were able to segment a continuous speech stream and start to encode the first syllables of the chunks. While the present study remains a toy experiment far from the complexity of a real-life environment, it reveals the underlying integration between successive functional processes computed in different neural structures that is at the core of infant learning.

## Materials and methods

### Participants

Participants were healthy-full-term neonates, with normal pregnancy and birth (GA > 38 weeks, Apgar scores ≥ 7/8 at 1/5 min, birthweight > 2.5 kg, cranial perimeter ≥ 33.0 cm), tested at the Port Royal Maternity (AP-HP), in Paris, France. The protocol was approved by the regional ethical committee for biomedical research (Comité de Protection des Personnes Region Centre Ouest 1, EudraCT/ID RCB: 2017-A00513-50), and the study was carried out according with relevant guidelines and regulations. Parents provided informed consent. 31 participants who provided enough data without motion artifacts were included (10 females; 1 to 4 days old; mean GA: 40.2 weeks; mean weight: 3475 g). Seven other infants were excluded from the analysis (3 due to excessive hair or cradle cap, 2 due to excessive motion artifacts, and 2 because the parents decided to interrupt the experiment).

### Stimuli

The stimuli were synthesized using the fr4 French female voice of the MBROLA diphone database^59^. Syllables had a consonant–vowel structure. Each phone had a duration of 125 ms and a constant pitch of 200 Hz. The streams were continuous with co-articulation and no pauses, and they were ramped up and down during the first and last 5 s to avoid the start and end of the stream might serve as perceptual anchors.

The structured streams consisted of a semi-random concatenation of the four tri-syllabic pseudo-words. Pseudo-words were concatenated with the only restrictions that the same word could not appear twice in a row, and the same two words could not repeatedly alternate more than two times (i.e., the sequence *W*_*k*_*W*_*j*_*W*_*k*_*W*_*j*_, where *W*_*k*_ and *W*_*j*_ are two words, was forbidden). The pseudo-words were created to avoid that specific phonetic features could help to segment the stream. Additionally, three different structured streams (lists) were used by modifying how the syllables were combined to form the Words (Table 1). Participants were randomly assigned and balanced between lists. The long learning stream lasted 180 s, each word appearing 60 times and each of the 12 possible part-words 18 to 21 times; the average TPs between words was 0.332 (SD = 0.017, range 0.310 to 0.361). The eight short structured learning streams lasted 30 s each, each word appearing 80 (8 × 10) times and each of the 12 possible part-words between 24 and 28 times; the average transitional probability between words was 0.325 (SD = 0.012, range 0.308 to 0.345).

The random stream was created using the same 12 syllables semi-randomly concatenated to achieve uniform TPs. The only restriction during the concatenation was that the same syllable could not appear twice in a row and that two syllables could not alternate more than two times (i.e., the sequence *S*_*k*_*S*_*j*_*S*_*k*_*S*_*j*_, where *S*_*k*_ and *S*_*j*_ are two syllables, was forbidden). Test words were tri-syllabic triplets presented in isolation.

### Procedure and data acquisition

Scalp electrophysiological activity was recorded using a 128-electrode net (Electrical Geodesics, Inc.) referred to the vertex with a sampling frequency of 250 Hz. Neonates were tested in a soundproof booth while sleeping or during quiet rest. The random streams and resting-state periods were sandwiching the learning and test parts to avoid a confound between time in the experiment and conditions, as changes in the vigilance state could induce. The study involved: (1) 60 s of resting-state; (2) 120 s of a random stream; (3) 180 s of a structured stream (4) 8 series of a 30 s of structured streams followed by 16 test-words (ISI 2–2.5 s) with 2.5 s of silence between the streams and the test-words; (5) 120 s of a random stream; (6) 60 s of resting state. The same 16 words (Table 1) were presented in each block in a random order and with a variable ISI between 2 and 2.5 s. The total duration of the recording session was ~ 20 mn.

### Data pre-processing

Data were band-pass filter 0.1–40 Hz and pre-processed using custom MATLAB scripts based on the EEGLAB toolbox 2021.0^60^, according to the APICE pre-processing pipeline^61^.

### Neural entrainment

The pre-processed data were resampled to 300 Hz to achieve an integer number of samples per triplet (225 samples in 0.75 s) and further high-pass filtered at 0.2 Hz. Then, data was segmented from the beginning of each phase into 0.75 s long segments. Segments containing samples with artifacts were rejected. Subjects who did not provide at least 6 segments per condition were not included. On average we retained 74% of the data during Resting (SD 17, range [31, 100]), 84% of the data during the Random (SD, 11, [47, 100]), and 87% of the data during the long and short Structured streams (SD 7, range [71, 100]).

#### Neural entrainment per condition

The 0.75 s epochs belonging to the same condition were reshaped into non-overlapping epochs of 7.5 s (10 triplets, 30 syllables), retaining the chronological order; thus, the timing of the steady state response. Data were referenced average and normalized by dividing by the standard deviation within an epoch. DSS, a technique based on spatial filters designed to remove stimulus-unrelated activity^62^, was applied, and the first 30 components of the first PCA and the first 6 of the DSS filter were retained (the pattern of results did not differ if DSS was not used). Next, data were converted to the frequency domain using the Fast Fourier Transform (FFT) algorithm, and the power and ITC were estimated for each electrode during each condition (Resting-state, Random, Structured). The power was computed as the power spectrum of the average response across trials. The ITC was computed as $ITC(f)=\frac{1}{N}\left|\sum_{i=1}^{N}{e}^{i\varphi (f,i)}\right|$, where N is the number of trials and φ(f,i) is the phase at frequency f and trial i. The ITC ranges from 0 to 1 (i.e., completely desynchronized activity to perfectly phased locked activity).

Finally, the SNR relative to the twelve adjacent frequency bins (six of each side corresponding to 0.8 Hz) was estimated for both measures. For the power the noise level was estimated at each frequency by assuming a power-law fit on the adjacent frequency bins log(P_estimate_(f)) = a + b*log(f). Then, the SNR for the power was SNR(f) = (log(P(f)) − mean(P_noise_(f)))/std(P_noise_(f)), where P_noise_(f) = log(P_estimate_(f)) − log(P). For the ITC the SNR was SNR(f) = (ITC(f) − mean(ITC_noise_(f)))/std(ITC_noise_(f)), where ITC_noise_(f) is the ITC over the adjacent frequency bins.

If no entrainment is present at a given frequency, then the SNR should be zero. Therefore, for statistical analysis, we compared the SNR for the power and ITC at the syllabic rate (4 Hz) and word rate (1.33 Hz) against zero using a one-tail t-test. P-values were corrected across electrodes by FDR.

#### Neural entrainment time course

The 0.75 s epochs were concatenated chronologically (1 min of RS, 2 min of Random, 3 min of long Structured stream, 4 min of short Structure blocks, 2 min of Random, and 1 min of RS). The same analysis than above was performed in sliding time windows of 2 min with a 1.5 s step.

### ERPs to test words

The pre-processed data were filtered between 0.5 and 20 Hz, epoched between [− 1.50, 3.25] s from the onset of the triplets. Epochs containing samples identified as artifacts were rejected. Subjects who did not provide at least 12 trials per condition were excluded. Data were reference averaged, normalized by dividing by its standard deviation, and baseline corrected by subtracting the average over the interval between 2.25 s from the onset of the previous word and the corresponding word. Trials were averaged by condition, and two contrasts were studied: (1) *ABx* (Words and Edge-words) vs. *BCx* (Part-words and Non-words) triplets; (2) triplets with heard transitions (Words and Part-words) vs. un-heard transitions (Edge-words and Non-words). The responses were compared using non-parametric cluster-based permutation analysis^43^ in two time windows: (1) [0, 0.5 s] to detect early effects only attributable to the encoding of the first syllables, and (2) [0.5, 2.75 s] to detect effects related to a TPs violation or to the triplets’ offset. A t-statistic with an alpha threshold of 0.05 was used for clustering; neighbor electrodes had a maximum distance of 3 cm (4.2 neighbors per channel on average); clusters had a minimum size of two, and 5,000 permutations were run to estimate the significance level. The quantification of the effect along test blocks was performed by computing the average difference between *ABx* and *BCx* conditions over the clusters. Data points were included for subjects and blocks when at least 3 out of 8 trials in both conditions were included.

### Adult behavioral experiment

33 French speaking adults were tested in an online experiment analogous to the infant study through the Prolific platform. All participants provided informed consent and received monetary compensation for their participation. The study was approved by the Ethical research committee of Paris Saclay University under the reference CER-Paris-Saclay-2019-063. The same stimuli as in the infant experiment were used. Participants first heard 3 min of familiarization with the Structured stream. Then, they completed eight sessions of re-familiarization and testing. Each re-familiarization lasted 30 s, and in each test session, all 16 possible test words were presented. Before starting the experiment, subjects were instructed to pay attention to an invented language because later, they would have to answer if different sequences followed to the structure of the language. During the test phase, subjects were asked to scale their familiarity with each test-word by clicking with a cursor on a scale from 1 to 6. One participant was excluded because (s)he always responded with a score of 1 or 2. Subjects were randomly assigned to one of the three lists.
